# Two New Steroidal Saponins from *Allium macrostemon* Bunge and Their Cytotoxity on Different Cancer Cell Lines

**DOI:** 10.3390/molecules14062246

**Published:** 2009-06-19

**Authors:** Hai-Feng Chen, Guang-Hui Wang, Qiang Luo, Nai-Li Wang, Xin-Sheng Yao

**Affiliations:** 1 Institute for Biomedical Research, Xiamen University, Xiamen 361005, China; E-mails: guanghui@xmu.edu.cn (G.H.W.), luoqiang20032004@yahoo.com.cn (Q.L.); 2 Department of Natural Products Chemistry, Shenyang Pharmaceutical University, Shenyang 110016, China; E-mail: wangnl@sz.tsinghua.edu.cn (N.L.W.)

**Keywords:** *Allium macrostemon* Bunge, steroidal saponins, cytotoxic activity

## Abstract

Two new steroidal saponins (**1** and **2**) were isolated from the dried bulbs of *Allium macrostemon* Bunge. Their structures were elucidated by the spectral data as 26-*O*-*β*-D-glucopyranosyl-5*α*-furost-25 (27)-ene-3*β*, 12*β*, 22, 26-tetraol-3-*O*-*β*-D-glucopyranosyl (1→2) [*β*-D-glucopyranosyl (1→3)]-*β*-D-glucopyranosyl (1→4)-*β*-D-galactopyranoside (**1**) and 26-*O*-*β*-D-glucopyranosyl-5*β*-furost-20 (22)-25 (27)-dien-3*β*, 12*β*, 26-triol-3-*O*-*β*-D-glucopyranosyl (1→2)-*β*-D-galactopyranoside (**2**), respectively. Their cytotoxic activities on several cancer cell lines (MCF-7, NCI-H460, SF-268 and HepG2) were tested. **1** showed special cytotoxity on SF-268, while **2** showed cytotoxity on NCI-H460 and SF-268 cell lines, respectively.

## Introduction

The dried bulbs of *Allium macrostemon* Bunge are well known as the traditional Chinese medicine “Xie bai”, which is used for treatment of throracic pain, stenocardia, heart asthma and diarrhea [1]. The main constituents of the ethanol extract of *Allium macrostemon* Bunge are steroidal saponins, which showed inhibitory effects on platelet aggregation [2] and degradation effects on blood serum peroxides proton [3], as well as the cytotoxic activity on different cancer cell lines [4,5]. This paper describes the isolation and structural elucidation of two new steroid saponins from “Xie bai” ethanol extract. Their cytotoxic activities against several human cancer cell lines, including MCF-7 (breast), NCI-H460 (lung), SF-268 (CNS) and HepG2 (liver), were also tested.

## Results and Discussion

The dried bulbs of *Allium Macrotemon* Bunge were extracted with 60% ethanol. The concentrated ethanol extract was passed through a Diaion HP-20 column eluting with a EtOH-H_2_O gradient. The 60% ethanol eluting fraction was collected and further fractionated by silica gel and octadecylsilanized silica gel and repeated Prep-HPLC to yield compounds **1** and **2**.

Compound **1** was obtained as an amorphous powder. The molecular formula was determined as C_57_H_94_O_30_ by the HR-ESIMS at *m/z* 1281.5723 [M+Na]^+^ (calcd. 1281.5728). The ^1^H-NMR of **1** showed three methyl signals at δ 0.63 (s), 1.36 (s) and δ1.61 (d, *J*= 6.8 Hz). Of all 57 carbon signals observed in ^13^C-NMR spectrum, 27 carbon signals (Me× 3, CH_2_× 12, CH× 8, C× 4), including a characteristic C_25_-C_27_ double bond carbon signals at δ 147.2 and 110.4. The ^1^H-NMR and ^13^C-NMR data indicated that **1** had the same sugar moiety as that of Macrostemonoside E [6] but a different aglycone. Comparison of the ^13^C-NMR data of the aglycone of **1** with those for Macrostemonoside E suggested differences at C-12, C-20, C-22, C-25 and C-27. The absence of characteristic C_2__0_-C_22_ double bond carbon at δ 152.4 and 103.7 and the presence of carbon signal at δ 110.9 revealed the presence of a hydroxyl group at C-22. In HMBC spectrum, the long-range correlations from δ 1.36 (H-18) to δ 79.3 (C-12) suggested the presence of hydroxyl at C-12 and correlations from olefinic proton signals at δ5.34, 5.70 (H-27) to δ 72.0 (C-26), 147.2 (C-25), 28.4 (C-24) suggested the presence of a C_25_-C_27_ double bond in the aglycone. In the NOESY spectrum, the absence of NOE correlation between proton signals at d 1.36 (H-18) and 3.58 (H-12) suggested the *β*-orientation of the C-12 hydroxyl and this was also confirmed by the comparison of C-12 carbon signal at δ 79.3 with Macrostemonoside G [7] which has the *β*-orientation of the C-12 hydroxyl at δ 79.6.

With the same NMR data of the sugar parts compared with Macrostemonoside E, the sugar moiety was finally unambiguously determined by acid hydrolysis and analysis of a combination of DEPT, ^1^H-^1^H COSY, HMQC, HMBC and TOCSY spectra. Then, the structure of **1** was determined as 6-*O*-*β*-D-glucopyranosyl-5*α*-furost-25(27)-ene-3*β*,12*β*,22,26-tetraol-3-*O*-*β*-D-glucopyranosyl (1→2) [*β*-D-glucopyranosyl (1→3)]-*β*-D-glucopyranosyl (1→4)-*β*-D-galactopyranoside.

Compound **2** was also isolated as an amorphous powder. Its molecular formula of C_45_H_72_O_19_ was deduced by positive-ion HR-ESIMS at *m/z* 939.4572 [M+Na]^+^ (calcd. 939.4566). The ^1^H-NMR spectrum contained three steroid methyl groups at δ 0.92 (3H, s), 0.99 (3H, s) and 2.00 (3H, s). These three single peak methyl proton signals, especially the signal shift downfield to δ 2.00 compared with that of signal at H-21 of **1**, revealed the presence of double bonds at C_25_-C_27_ and C_20_-C_22_. Comparison of the ^13^C-NMR spectrum of **2** with that of Macrostemonoside G [7] showed considerable structural similarity. However, the molecular formula of **2** was lower by 18 units (one H_2_O) than that of Macrostemonoside G and the difference were recognized in the carbon signals from the ring E portion. The ^13^C-NMR spectrum showed that the carbon signals of C-16, C-17, C-20, C-22 and C-24 of **2** were shifted downfield by approximately + 3.3, + 0.9, + 63.2, + 31.1 and + 2.8 ppm, respectively, while the carbon signal of C-21 and C-23 shifted to higher field by - 3.3 and -13.1 ppm comparing with those of Macrostemonoside G, which suggested the presence of a double bond between C-20 and C-22. This was also confirmed by long-range correlations between the proton signal at δ 0.92 (H-21) and carbon signals at δ 104.8 (C-20) and 151.7 (C-22) in the HMBC spectrum. The HMBC correlation between the proton signal at δ 0.92 (CH_3_-18) and the carbon signal at δ 78.7 (C-12) indicated a hydroxyl group at C-12. In the NOESY spectrum, the β-orientation of the C-12 hydroxyl group was inferred due to the absence of NOE correlation between the proton signals at δ 0.92 (H-18) and 3.53 (H-12). The triglycoside moiety of **2** was shown to be the same as that Macrostemonoside G and the structure of **2** was assigned as 26-*O*-*β*-D-glucopyranosyl-5*β*-furost-20 (22)-25 (27)-dien-3*β*,12*β*,26-triol-3-*O*-*β*-D-glucopyranosyl (1→2)-*β*-D-galactopyranoside.

The *in vitro* cytotoxicity of compounds **1** and **2** against various cancer cell lines was evaluated by MTT assay. IC_50_ values were calculated by the LOGIT method (Table 1). Compound **1** showed mild cytotoxity, especially towards the SF-268 cell line with IC_50_ values of 35.2 μM and compound **2** showed mild cytotoxity, especially to the SF-268 and NCI-H460 cell lines, with IC_50_ values of 25.7 and 35.4 μM, respectively. Comparison of the structure and the cytotoxic activity of **1** and **2** with those steroidal saponins reported previously [5] suggests that the presence of a C_25_-C_27_ double bond and C-12 hydroxyl in the steroidal pentaglycoside aglycone contribute to the cytotoxicity in the SF-268 cell line and a C_20_-C_22_ double bond in the steroidal triglycoside aglycone containing a C-12 hydroxyl contribute to the selective cytotoxicity to the SF-268 and NCI-H460 cell lines, respectively.

**Figure 1:**
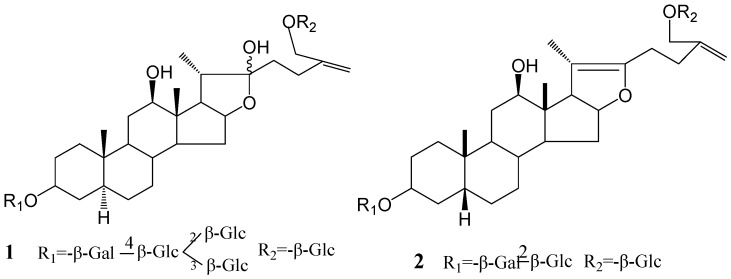
The structures of compounds **1** and **2**.

**Figure 2:**
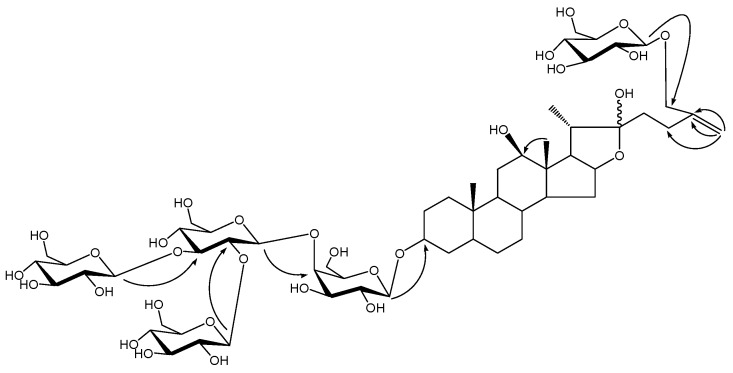
The key HMBC of compound **1**.

**Table 1:** *In vitro* cytotoxic activity of compounds **1** and **2** on cancer cell lines^a^.

Compounds	Cell lines IC_50_(μM)			
	NCI-H460	SF-268	MCF-7	HEPG2
1	>100	35.2±1.02	>100	>100
2	25.7±0.62	35.4±0.71	>100	>100

^a^ Data shown are the mean IC_50_ of three independent experiments ±SD.

**Table 2:** ^1^H-NMR and ^13^C-NMR data of compound **1** (C_5_D_5_N) ^a^.

Position	δ_C_	δ _H_	Position	δ_C_	δ _H_
1	37.1(t)	1.54, 0.79(o)	C3 Gal-1	102.4(d)	4.81(d, *J*= 7.8)
2	29.8(t)	1.96, 1.22(o)	2	73.1(d)	4.44(o)
3	75.1(d)	4.07(o)	3	75.5(d)	4.23(o)
4	34.7(t)	1.78, 1.31(o)	4	80.1(d)	4.62(m)
5	44.6(d)	0.83(o)	5	76.1(d)	4.14(o)
6	28.9(t)	1.14, 1.09(o)	6	60.5(t)	4.65,4.23(o)
7	32.2(t)	1.49, 0.48(o)	(Inner)Glc-1	105.0(d)	5.51(d, *J*= 7.8)
8	34.3(d)	1.46(o)	2	81.4(d)	4.35(o)
9	53.5(d)	0.63(o)	3	88.5(d)	4.21(o)
10	35.7(s)	----	4	70.7(d)	3.81(o)
11	31.6(t)	1.83, 1.46(o)	5	77.8(d)	4.16(o)
12	79.3(d)	3.58(m)	6	62.3(t)	4.56(o)
13	46.7(s)	----	3-Glc-1	104.5(d)	5.26 (d, *J*= 7.8)
14	55.0(d)	1.14(o)	2	75.2(d)	4.05(o)
15	32.4(t)	2.06, 1.55(o)	3	78.6(d)	3.81(o)
16	81.2(d)	5.07(o)	4	70.8(d)	4.26(o)
17	63.7(d)	2.33 (t, *J*= 8.2)	5	77.5(d)	3.82(o)
18	11.3(q)	1.36(s)	6	62.7(t)	4.14,4.28(o)
19	12.2(q)	0.63(s)	2-Glc-1	104.8(d)	4.75 (d, *J*= 7.8)
20	41.6(d)	2.41(m)	2	75.1(d)	4.10(o)
21	15.6(q)	1.61 (d, *J*= 6.8)	3	78.6(d)	4.24(o)
22	110.9(s)	----	4	71.6(d)	4.20(o)
23	38.0(t)	2.45(o)	5	78.4(d)	3.95(o)
24	28.4(t)	2.76(m)	6	62.8(t)	4.18,4.05(o)
25	147.2(s)	-----	C-26 Glc-1	103.9(d)	5.15 (d, *J*= 7.8)
26	72.0(t)	4.52(o)	2	75.2(d)	4.03(o)
27	110.4(t)	5.34(s); 5.70(s)	3	78.6(d)	4.05(o)
			4	71.5(d)	4.21(o)
			5	78.5(d)	4.18(o)
			6	63.0(t)	4.52,4.23(o)

^a^Recorded on a Bruker-400 NMR spectrometer (100 MHz for ^13^C).

**Table 3:** ^1^H-NMR and ^13^C-NMR data of compound **2** (C_5_D_5_N) ^a^.

Position	δ_C_	δ _H_	Position	δ_C_	δ _H_
1	31.0(t)	1.79, 1.95 (o)	C3 Gal-1	102.5(d)	4.90 ( d, *J*= 7.6)
2	26.8(t)	2.00, 1.49 (o)	2	81.8(d)	4.65 (o)
3	75.5(d)	4.33 (o)	3	75.2(d)	4.23 (o)
4	31.0(t)	1.79, 1.98 (o)	4	69.9(d)	4.56 (o)
5	36.8(d)	2.17 (o)	5	76.6(d)	4.00 (o)
6	27.1(t)	1.21, 1.91 (o)	6	62.2(t)	4.35, 4.42 (o)
7	26.8(t)	1.99, 1.51 (o)	Glc-1	106.0(d)	5.29 (d, *J*= 7.6)
8	34.3(d)	1.45 (o)	2	76.9(d)	4.05 (o)
9	39.5(d)	1.46 (o)	3	78.1(d)	4.15 (o)
10	35.3(s)	------	4	71.8(d)	4.35 (o)
11	31.6(t)	2.50 (o)	5	78.4(d)	3.83 (o)
12	78.7(d)	3.53 (m)	6	62.9(t)	4.32, 4.51 (o)
13	50.0(s)	------	C26 Glc-1	103.8(d)	4.93 (d, *J*= 7.6)
14	53.5(d)	3.24 (m)	2	75.2(d)	4.00 (o)
15	31.2(t)	1.87 (o)	3	78.6(d)	4.21 (o)
16	84.6(d)	4.92 (o)	4	71.9(d)	4.22 (o)
17	64.6(d)	2.92 (d, *J*= 10.4)	5	78.5(d)	3.90 (o)
18	9.5(q)	0.92 (s)	6	62.9(t)	4.37, 4.57 (o)
19	23.9(q)	0.99 (s)			
20	104.8(s)	------			
21	12.3(q)	2.00 (s)			
22	151.7(s)	------			
23	24.9(t)	2.48 (o)			
24	31.2(t)	2.54 (o)			
25	146.3(s)	------			
26	71.8(t)	4.43, 4.00 (o)			
27	111.6(t)	5.10, 5.39 (o)			

^a^ Recorded on a Bruker-400 NMR spectrometer (100 MHz for ^13^C).

## Experimental

### General

Melting points were determined with a Yanaco MP-S_3_ micro-melting point apparatus and are uncorrected. Optical rotations were obtained on a P-1020 digital polarimeter (JASCO Corporation). IR spectra were measured on a Shimadzu FT/IR-8400 spectrometer. 1D and 2D NMR spectra were taken on a Bruker AV-400 spectrometer in C_5_D_5_N solution (at 400 MHz for ^1^H-NMR) . ESIMS spectra were acquired using a Bruker Esquire 2000 mass spectrometer. Column chromatography was carried out on Diaion HP-20 (Mitsubishi Kasei), silica gel (200-300 mesh, Qingdao Factory of Marine Chemical Industry, Qingdao, China) and ODS (40-63μm, Merck). TLC analyses were taken on Silica gel 60F_254_ (Qingdao Factory of Marine Chemical Industry, Qingdao, China) and the spots were detected spraying with Ehrlich reagent and heating. Preparative HPLC was performed using an ODS column (250× 20mm, 10μm, SHIMADZU Pak; Detector: RID). 3-(4,5-dimethyl-thiazol-2-yl)-2,5-diphenyl-tetrazolium bromide (MTT), were purchased from Sigma (St. Louis, MO, USA). RPMI-1640 medium, fetal bovine serum (FBS) and trypsin-EDTA solution (1X) were obtained from GIBCO-BRL (Grand Island, NY, USA).

### Plant material

The bulbs of *Allium Macrostemon* Bunge were purchased from Liaoning Province, P. R. China, and identified by Professor Qishi Sun (Department of Pharmacognosy, Shenyang Pharmaceutical University). The voucher specimen (No.203554) has been deposited at the Department of Natural Product Chemistry, Shenyang Pharmaceutical University, P. R. China.

### Extraction and isolation

The dried bulbs of *Allium Macrotemon* Bunge (6 kg) were extracted twice with 60% ethanol for 2 hours each. The alcoholic extract was concentrated under reduced pressure, suspended in water and then passed through Diaion HP-20 column using EtOH-H_2_O gradient system (0-100%). The 60% EtOH eluate fraction (100 g), which was subjected to silica gel column chromatography with CHCl_3_-MeOH-H_2_O (9:1:0.1; 8:2:0.2; 7:3:0.5; 6:4:0.8) and MeOH finally, gave nine fractions. Fraction 6 was further purified by ODS column chromatography eluting with MeOH-H_2_O (3:7; 4:6; 5:5) and repeated Rp-18 HPLC preparation to yield **1** (10.2 mg) and **2** (15.5 mg).

*Compound* **1**: An amorphous powder, mp 205-206°C; ${\left[\alpha \right]}_{D}^{21}$ –27.4° (H_2_O, *c* 0.10); HR-ESIMS (positive mode) at *m/z* 1281.5723 [M+Na]^+^ (calcd. 1281.5728); ESIMS (positive mode) at *m/z* 1,281 [M+ Na]^+^, 1,263 [M+ Na- H_2_O]^+^, 1,119 [M+ Na- 162]^+^, 1,101 [M+ Na- H_2_O- 162]^+^, 939 [M+ Na- H_2_O-162× 2]^+^, 777 [M+ Na- H_2_O- 162× 3]^+^; ESIMS (negative mode) at *m/z* 1,257 [M- H]^-^, 1,095 [M- H- 162]^-^, 933 [M-H-162×2]^-^, 771 [M- H- 162×3]^-^, 609 [M- H- 162× 4]^-^; IR ν_max_ (KBr) cm ^-1^: 3,420 (OH), 2,938 (CH), 1,000-1,100; ^1^H-NMR (C_5_D_5_N) and ^13^C-NMR data see Table 2.

*Compound **2***: An amorphous powder, mp 175-177°C; ${\left[\alpha \right]}_{D}^{21}$ 4.8° (H_2_O, *c* 0.08); HR-ESIMS (positive mode) at *m/z* 939.4572 [M+Na]^+^ (calcd. 939.4566). FABMS (n= 1) at *m/z* 917 [M+ H]^+^, 755 [M+ H- 162]^+^, 737 [M+ H- 162- H_2_O]^+^, 593 [M+ H- 162× 2]^+^, 575 [M+ H- 162× 2- H_2_O]^+^, 431 [M+ H- 162×2- 162]^+^; IR ν_max_ (KBr) cm ^-1^: 3,410 (OH), 2,924 (CH), 1,010-1,120; ^1^H-NMR (C_5_D_5_N) and ^13^C-NMR data see Table 3.

### Acid hydrolysis of saponins

Each saponin (5 mg) was heated in an ampoule with aq. 15% HCl (5 mL) at 110 °C for 2 h. The aglycon was extracted with dichloromethane three times and the aqueous residue was evaporated under reduced pressure. Then, pyridine (1 mL) and NH_2_OH·HCl (2 mg) were added to the residue, and the mixture was heated at 100 °C for 1 h. After cooling, Ac_2_O (0.5 mL) was added and the mixtures were heated at 100 °C for 1 h. The reaction mixtures were evaporated under reduced pressure, and the resulting aldononitrile peracetates were analyzed by GC-MS using standard aldononitrile peracetates as reference samples.

### Cell culture 

MCF-7, NCI-H460, SF-268 and HepG2 cells were maintained in RPMI 1640 (Gibco BRL) containing 10% FBS (Gibco), 2 mg/mL sodium bicarbonate, 100 µg/ml penicillin sodium salt and 100 µg/mL streptomycin sulfate. Cells were grown to 70% confluence, trypsinized with 0.05% trypsin-2 mM EDTA, and plated for experimental use. In all experiments, cells were grown in RPMI-1640 medium with 10% FBS for 24 hr prior to treatment.

### Cytotoxicity assay

1.0×10^4^ MCF-7, NCI-H460, SF-268 and HepG2 cells were seeded in 96 well tissue culture plates and treated with the two compounds on different concentration for 48 h. MTT (3-[4,5-dimethylthiazol-2-yl]-2,5-diphenyltetrazolium bromide) reagent (5 mg/mL in PBS, 10 μL) was added to each well and incubated for 4 h. After that, the suspended liquid was poured out and DMSO (100 μL) added to each well and swirled gently. Finally, the plate cover was removed and the absorbance in each well measured at 570 nm in a micro titer plate reader [8].
